# Obliteration of bacterial growth and biofilm through ROS generation by facilely synthesized green silver nanoparticles

**DOI:** 10.1371/journal.pone.0181363

**Published:** 2017-08-03

**Authors:** Shariq Qayyum, Mohammad Oves, Asad U. Khan

**Affiliations:** 1 Medical Microbiology and Molecular Biology Laboratory Interdisciplinary Biotechnology Unit, Aligarh Muslim University, Aligarh, India; 2 Center of Excellence in Environmental Studies (CEES), King Abdulaziz University, Jeddah, Kingdom of Saudi Arabia; VIT University, INDIA

## Abstract

*Mangifera indica* inflorescence aqueous extract was utilized for production of green AgNPs. Synthesized AgNPs were characterized by UV-vis spectrophotometry, XRD, TEM, FESEM and particles size analyzer. AgNPs showed minimum inhibitory concentrations (MICs) of 8 μg ml^-1^ and 16 μg ml^-1^ for Gram negative *(K*. pneumoniae, *P*. *aeruginosa* and *E*. *coli*) and Gram positive (*S*. *mutans* and *S*. *aureus*) strains, respectively which was relatively quite low compared to chemically synthesized silver nanoparticles. AgNPs inhibited 80% and 75% biofilms of *E*. *coli* and *S*. *mutans* respectively as observed quantitatively by crystal violet assay. Qualitative biofilm inhibition was observed using SEM and CLSM. AgNPs adsorbed catheter also resisted the growth of biofilm on its surface displaying its possible future applications. AgNPs interaction with bacteria lead to bacterial membrane damage as observed by SEM and TEM. The membrane damage was confirmed by detecting leakage of proteins and reducing sugars from treated bacterial cells. AgNPs generated ROS on interaction with bacterial cells and this ROS production can be one of the possible reasons for their action. AgNPs exhibited no toxic effect on the cell viability of HeLa cell line.

## Introduction

Biofilms are complex colony set up of microorganisms which enable them to escape various harsh conditions. It contains a complex matrix structure mainly made of exopolysaccharide and bacterial cells remain embedded in the matrix. This acts a shield for these microbes against many unfavorable environmental including antibacterial agents. Biofilm has been related with numerous diseases, i.e; otitis media, endocarditis, chronic prostatitis, cystic fibrosis, periodontitis and other biomedical device related infections [1]. Modification of functional groups and reduced penetration of antimicrobials by biofilm components enhances the antibiotic resistance.

Silver nanoparticles have emerged as strong antimicrobial agents. Antibacterial, antifungal, anti-inflammatory and anti-viral activities of silver nanoparticles (AgNPs) have already been reported which showed biomedical their significance [2, 3]. Numerous methods have been described for the synthesis of AgNPs e.g., chemical reduction, thermal decomposition, electrochemical, radiation, sonochemical and microwave assisted processes and recently via green chemistry route [4]. Green synthesis is an emerging technology which exhibited its significance over the chemical and physical methods. The use of plants extracts for nanoparticles synthesis has demonstrated its advantages over other biological processes because it eliminates the intricate process of maintaining cell cultures and can also be suitably scaled up for large-scale synthesis of nanoparticles under non- aseptic environment. Palanisamy *et al*. (2014) has previously described antibiofilm properties of chemically synthesized AgNPs against *P*. *aeruginosa* [5]. Chemically synthesized nanoparticles raises toxicity issues for using in human systems. This toxicity also arises because of the toxic reagents used in the synthesis of nanoparticles and remains with particles as adsorbent.

The mango inflorescence taken in this study contains number of beneficial organic compounds such as gallic acid, ethyl gallate, methyl gallate, n-propyl gallate, n-pentylgallate, n-octylgallate, 4-phenyl gallate, 6-phenyl-n-hexyl gallate and dihydrogallic acid and its essential oil containing linalool, elemene, ocimene, nerol, humulene and many others coumoinds that are double bonded and have contributed for the reduction of silver ions and antibacterial nature of these nanoparticles. In another study has mentioned presence of naturally occurring steroids, triterpenes, phenolic, flavonoids and polyphenol was major component in mango inflorescence [6]. Many of the above mentioned compounds have been reported to contain antibacterial activities which may have enhanced the antibacterial activity of biosynthesized AgNPs [7, 8].

In this article, an effort has been made to use a safe and ecofriendly procedure to synthesize antimicrobial and antibiofilm silver nanoparticles using aqueous extract of *Mangifera indica* inflorescence. Antibiofilm action of these nanoparticles is quite important as biofilm may be reservoir for pathogenic organisms and source of infections. Our other objective is to figure out the mechanism of action of green AgNPs on the bacterial cells.

## Materials and methods

### Chemicals, plant material and microbial cultures

Analytical-grade AgNO_3_ was purchased from Qualigens. All analytical reagents and microbial media were bought from Hi-Media (India). For the synthesis of silver nanoparticles healthy inflorescence of mango trees (*Mangifera indica*) was collected from the campus premises of Aligarh Muslim University, Aligarh, India and was identified by the Department of Botany. The bacterial and fungal cultures were acquired from Interdisciplinary Biotechnology Unit’s departmental culture collection, Aligarh Muslim University, Aligarh, India. The used cultures are *Pseudomonas aeruginosa (P1)*, *Escherichia coli (ATCC-25922)*, *Klebsiella pneumoniae (ATCC-700603)*, *Streptococcus mutans (MTCC-497)* and Staphylococcus aureus *(ATCC-29213)*. Glassware used in this study was washed with aqua regia water and rinsed multiple times with double distilled water.

### Preparation of inflorescence extract

**Fig 1:**
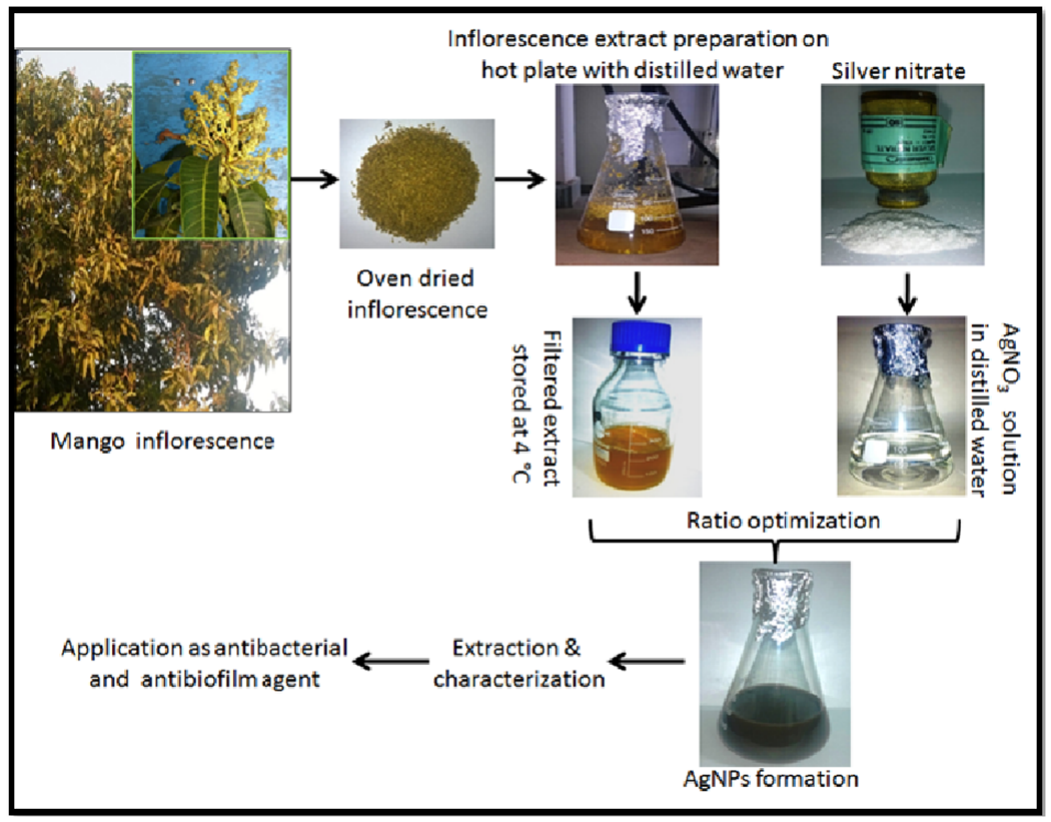
Graphical presentation of green silver nanoparticles biosynthesis.

Inflorescence of *Mangifera indica* was collected from the young tree of *Mangifera indica*. The inflorescence was washed many times with double distilled water (DDW) to eliminate the dust particles and pollutants from surface before oven-drying (80°C). The oven drying completely evaporated the remaining moisture in the samples. Then 20 g processed inflorescence was cut into smaller pieces and boiled in a 500 ml Erlenmeyer flask along in 200 ml of sterile DDW for 5 mins. The aqueous extract was then filtered using Whatman No. 1 filter paper (Maidstone, UK). Residual particles after filtration were sedimented using centrifugation at 5000 rpm for 5 min. The extract was stored at 4°C and was further used for biosynthesis of silver nanoparticles from silver (Fig 1) [9 & 10].

### Preparation of chemically synthesized and biogenic AgNPs

In a typical reaction procedure, 5 ml of inflorescence extract was mixed with 100 ml of 1 mM aqueous AgNO_3_ solution, with continuous stirring at room temperature. The changes in color of the mixture was observed and the spectra were recorded from 0 min to 24 hours. AgNO_3_ concentrations was changed from 1 to 5 mM in the reaction mixture. UV-visible (UV–vis) spectra was observed for surface plasmon resonance (SPR) band for the formation of silver nanoparticles. The green AgNPs synthesized in the process were harvested by centrifugation of the solution at 15,000 rpm for 45 min. To remove excess silver ions present, the pellets were washed three times with deionized water. It was then lyophilized and stored in screw-capped vials under ambient conditions for further characterization and application [11]. Turkevich method was used to synthesize the chemically made AgNPs (Characterization data not shown) [12].

### Characterization of green AgNPs

#### UV–vis spectroscopy and Nanophox spectra analysis

The prepared AgNPs were primarily characterized by UV-vis spectroscopy, which has proved to be a very convenient method for the analysis of AgNPs. UV-vis spectra of AgNPs were taken using Systronics double beam spectrophotometer (Model no. M2202 India) worked with path length of 10 mm and at a resolution of 2 mm using quartz cells. Blanks were prepared with silver nitrate solution in double distilled water. The conversion of silver ions into AgNPs was examined by measuring the UV–vis spectrum of the reaction mixture from 0 min to 24 h in the wavelength ranging from 300–800 nm. In addition, stability of nano-suspension was analyzed by Nanophox particle size analyzer. Later, analyses of biogenic nanoparticles distribution and stability in solution were studied using the Nanophox [13].

#### Field emission scanning electron microscopy

Morphology of green AgNPs was examined by field emission scanning electron microscopy (FESEM), Hitachi SU6600. Small amount of the sample was placed on a carbon coated copper grid and prior to visualization grids were allowed to dry at room temperature [14].

#### Transmission electron microscopy

JEM 1101 transmission electron microscope (JEOL, Tokyo, Japan) was used to study the size and shape of the AgNPs. Ethanol was used to disperse the sample before placing it on a carbon-coated copper TEM grid, and the images were obtained by operating at an accelerating voltage of 120 Kv [13].

#### X-ray diffraction analysis

Powdered and lyophilized samples were set into XRD analysis for studying their crystalline nature and the diffraction patterns were recorded in the scanning mode on an X’pert Pro diffractometer (PANalytical, Almelo, the Netherlands) operated at 40 kV and with a current of 40 mA, with Cu/kα radiation (λ = 1.5418 Å) in the range of 20°–80° in 2θ angles [15].

#### FTIR spectra analysis

RXI FTIR spectrometer with KBr beamsplitter was utilized to recognize the biogroups that are attached on the surface of AgNPs. Free biomass residue was removed from the reaction mixture by centrifugation at 15,000 rpm for 30 min and resulted pellets were dissolved in 20 ml sterile double distilled water and cyclo-mixed for 10 min on vortex mixer. Centrifugation and re-dispersing process was repeated three times. The FTIR spectra of AgNO_3_, powdered inflorescence mass and AgNPs were recorded as a KBr pellet at a resolution of 4 cm^-1^ in the wave number region of 500–4000 cm^-1^ [16].

### Determination of minimum inhibitory concentration (MIC) and minimum bactericidal concentration (MBC)

The MIC and MBC of the biosynthesized nanoparticles along with the chemically synthesized AgNPs were tested against Gram positive and Gram negative bacteria by micro-dilution method following CSLI guidelines. All strains of bacteria were treated with increasing concentrations of the nanoparticles ranging from 0.125 μg ml^-1^ to 1024 μg ml^-1^ in a series of two fold dilutions. The lowest concentration that totally inhibits visible bacterial or fungal growth was considered as MIC. MBC was determined by sub-culturing the test dilutions on a LB agar plates and incubating for 24 h. All these determinations represent the mean for three independent experiments [17].

### Effect on growth of *E*. *coli* and *S*. *mutans*

Growth of *E*. *coli* and *S*. *mutans* was observed in presence of sub MIC concentrations of AgNPs. Overnight cultures of *E*. *coli* and *S*. *mutans* were inoculated into tubes to obtain a final inoculum of 1.6 × 10^4^ CFU ml^−1^ followed by the addition of AgNPs. The tubes were incubated at 37°C. Growth was observed spectrophotometrically (UV mini 1240, UV-VIS Spectrophotometer Shimadzu, New Delhi, India) by taking the absorbance of the culture at 600 nm for 24 h. All determinations were performed as triplicates using untreated growth controls [18].

### Biofilm quantification and visualization of biofilm architecture using crystal violet assay, scanning electron microscopy and confocal microscopy

#### Crystal violet assay quantification of biofilms

Biofilm formation assay was carried out in flat bottomed 96-wells microtitre plates. Over-night culture of all bacteria was inoculated in fresh LB medium and incubated at 37°C under aerobic condition to the mid- log phase (1.0 OD_600_). The cultures were then diluted to 1:100 in pre-warmed media. Bacterial suspension (200 μl) was added in the wells of plates with varying concentrations of the nanoparticles (0.125 μg ml^-1^ to 1024 μg ml^-1^). The un-inoculated media treated with nanoparticles was taken as negative control and media alone was used as blank control. After inoculation, all the plates were incubated at 37°C for 24 h. As, all strains taken in this study grows optimally at 37°C. The culture was then decanted and the plates were gently washed thrice with 200 μl of sterile distilled water to remove planktonic and other loosely bound cells. The attached bacterial cells were stained with 50 μl of 0.1% crystal violet for 15 min. After rinsing twice with 200 μl of sterile water, the bound dye was removed from the stained cells using 200 μl of 99% ethanol. Plates were then shaken for 5 min, so that all dye can be released form the cells. Biofilm inhibition was quantified by measuring optical density of the suspension at 630 nm using a microplate reader (BIORAD iMark TM Microplate Reader, India) [13].

#### Scanning electron microscopy of biofilms

Sub-MIC concentrations of AgNPs were used to treat the bacterial cells. Untreated bacterial cells of *E*. *coli* and *S*. *mutans* were taken as control. The wells were inoculated (10^5^–10^6^ CFU ml^−1^) and incubated at 37°C for 24 h. The coverslips were removed after 24 h and washed three times in sterile PBS. 2% formaldehyde and 2.5% glutaraldehyde was used to fix the samples after washing. The samples were rinsed three times with PBS after fixing, followed by an ethanol dehydration series. Samples were then completely dried, coated with gold, and observed using a scanning electron microscope [13].

#### Confocal microscopy of biofilms

Sub-MIC concentrations of AgNPs treated samples and untreated samples were taken for the confocal imaging. The inoculation was done in the covered glass bottom confocal dishes (Genetix Biotech Asia) with a dish size of 35 mm, 22 mm coverglass, 9.4 cm^2^ growth area and a working volume of 3 ml. The dishes were inoculated with 2(10^5^–10^6^ CFU m l^−1^) and incubated at 37°C for 24 h. After incubation the dishes were washed with sterile PBS and sequentially treated with SYTO-9 (5 μM; excitation and emission wavelength; 488 nm, 498 nm) and propidium iodide (PI) (0.75 μM; excitation and emission wavelength; 536 nm, 617 nm). The stained confocal discs were then scanned for the biofilm visualization using FluoView FV1000 (Olympus, Tokyo, Japan) confocal laser scanning microscope equipped with argon and HeNe lasers [18].

### Protein and reducing sugar leakage test

AgNPs and bacterial cells were added into 10 ml media with final concentration reaching MICs values of green AgNPs. Bacterial cell rupture leads to release of intracellular biomolecule like reducing sugars and protein. The increase in concentration of these biomolecules after treatment is a strong indicative of bacterial membrane damage. The bacterial cell count was kept at 10^9^ CFU ml^-1^ for detecting the leakage of reducing sugars and proteins from cells. Control experiments did not include AgNPs in the media. The cultures were incubated at 37±2°C with shaking at 150 rpm. Samples were evaluated at 0 hour and 4 h incubation for reducing sugar and protein leakage respectively. After incubation samples were centrifuged at 12,000 rpm, the supernatant liquid was frozen at −80°C immediately, and then the concentrations of reducing sugars and proteins were calculated [19].

### Electron microscopic analysis of interaction of AgNPs and bacterial cells

Interaction of *S*. *mutans* and *E*. *coli* cells with AgNPs was observed using transmission electron microscopy. Overnight grown bacterial cultures treated with sub-MICs concentration of AgNPs were taken for analysis. TEM was performed with a JEM 1101 transmission electron microscope (JEOL, Tokyo, Japan) to verify the interaction between two. The images were obtained by operating machine at an accelerating voltage of 180 kV for studying sample dispersed on a carbon-coated copper TEM grid. The particle elemental identity was known using EDAX technique.

### Reactive oxygen species (ROS) studies

#### Detection of ROS production

Dye 2′, 7′-dichlorofluorescein diacetate (DCFH-DA) was used to detect the ROS produced by green AgNPs. The number of bacterial cells was adjusted to 10^5^ CFU ml^−1^ with sub-MIC concentration of AgNPs. Cultures were incubated with 5μM DCFH-DA at 37°C for 4 h. After incubation supernatant was collected by centrifugation at 4°C for 30 min at 8,000 g. Fluorescence excitation wavelength of 485 nm and an emission wavelength of 525 nm was used to detect the ROS produced in the sample [18].

#### Visualization of ROS in biofilms

ROS generation in AgNPs treated biofilms were visualized by DCFH-DA (10 μM; excitation and emission wavelength; 495 nm and 520 nm) fluorescent dyes molecular probe [20]. Biofilms were treated with the MIC concentration of the AgNPs and incubated for 1h. The stained bacterial biofilm was observed with a FluoView FV1000 (Olympus, Tokyo, Japan) confocal laser scanning microscope equipped with argon and HeNe lasers.

#### Plasmid DNA cleavage activities

Commercially available bacterial plasmid pBR322 was used for this assay. Cleavage experiments of plasmid was carried out in presence of various free radical quenchers such as dimethyl sulfoxide (DMSO) (1.0 mM), tetra-butyl alcohol (TBA; 1.0 mM), sodium azide (NaN_3;_ 1.0 mM) and superoxide dismutase (SOD; 25 units). Plasmid DNA (1μg) was treated with AgNPs in presence of each free radical inhibitor and after that treated solution samples was incubated for 1 hour at 37°C. After incubation, each sample was treated with loading buffer (25% bromophenol blue, 0.25% xylene cyanol and 30% glycerol). The electrophoresis of these samples was carried out in 1% agarose gel containing Tris-HCl buffer at 60 V for 1 hour.

### In vitro biofilm assay on catheter

Non-toxic, pyrogen free, disposable, X-ray opaque line and sterile catheter (HARSONS) of 40 cm length was purchased from the local medical store. Catheter was cut into small pieces of 5 cm by an autoclaved medical blade under sterilized conditions. Catheter pieces were placed in dispersive colloidal AgNPs suspension having concentration of 100 μg ml^-1^ and kept in 37°C for 6 h in water bath sonicator. Sonication forces high speed AgNPs collision with catheter surface causing there embedding to the surface. Six-well microtitre plate was then used to grow biofilm onto the coated and uncoated catheter surface.

### Cell line toxicity assay

RPMI 1640 (Sigma Aldrich) was selected as the culture media to grown and maintain the HeLa cell line (HeLa cell line obtained from NCCS Pune, Maharashtra) containing 10% heat-inactivated fetal calf serum and antibiotic and antimycotic solution (Sigma Aldrich). 96- Well plate with cell concentration of 5 x 10^3^ cells per well was taken and incubated for 24 h at 37°C for cell multiplication. After this time period cells were exposed to various concentration of AgNP’s and incubated again for 48 h. Cell proliferation was measured by adding 20 μl of MTT (Sigma Aldrich) dye (5 mg ml^-1^ in phosphate-buffered saline) per well. The plates were incubated further for 4 h at 37°C in a humidified chamber containing 5% CO_2_. Formazan crystals formed due to reduction of dye by viable cells in each well were dissolved in 150 μl dimethyl sulfoxide and absorbance was recorded at 570 nm. Cell viability was measured in terms of absorption values and IC_50_ was calculated as 50% cell viability of control group [15].

### Statistical analysis

All experiments were performed in triplicate sets. For all assays including biochemical and other assay, data is presented along with mean ± standard deviation (SD). The values were calculated as the mean of individual experiments in triplicate and compared with those of the control groups. Student’s test was exploited for calculating differences between two mean values. Data with *p*-values < 0.05 were considered statistically significant and are reported.

## Results

### Characterization of AgNPs

#### AgNPs characterization using UV-vis spectroscopy and Nanophox particle size analysis

**Fig 2:**
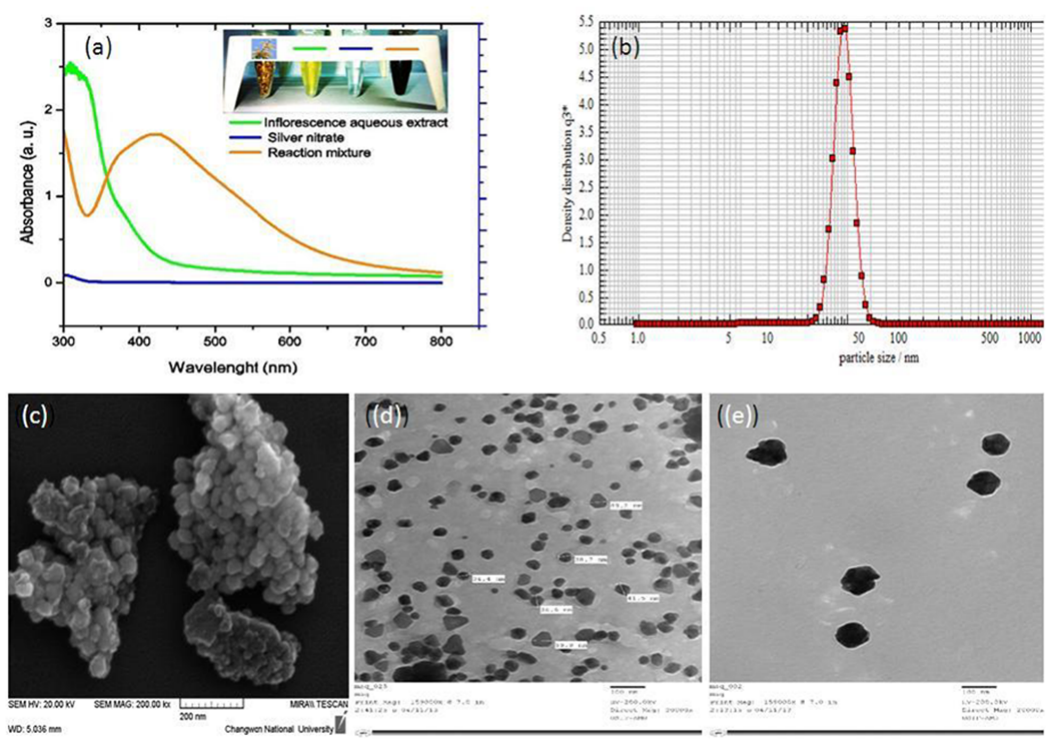
Characterization of green AgNPs (a) UV-vis spectra (b) Nanophox particles size analyzer graph (c) FE-SEM image (d) and (e) TEM images.

The biogenic AgNPs were characterized by UV-vis spectroscopy based on SPR peaks. The UV-vis absorption spectra of the AgNPs were measured in the range of 300–800 nm. Surface plasmon peak located at 423 nm which strong and broad indicative for the AgNPs formation (Fig 2a). The strong SPR located at 423 nm clearly indicates the synthesis of AgNPs, which were extremely stable, with no evidence of flocculation and aggregation of the particles even after 3 months. In order to understand the average size of synthesized AgNPs, a size-distribution analysis was performed using nanoparticle size analyzer (Nanophox) in aqueous solution. Fig 2b shows that the size of the particle was ranging between 30 and 70 nm with an average particle size of 40 nm.

#### Field emission scanning electron microscopy and transmission electron microscopy for AgNPs shape and size

The 3-D shape of the particles was evaluated by FESEM. The size of the particles was observed to be ranged as 30–70 nm with irregular shape as seen the image (Fig 2c). As, visualized by TEM images, it was seen that the AgNPs size was in accordance with the other results and size of particles was between 30 nm to 70 nm with an average size of ~40nm. The images revealed that the nanoparticles were monodispersed and with no aggregation even after 3 months (Fig 2d & 2e).

#### Crystallinity confirmation by XRD Analysis

**Fig 3:**
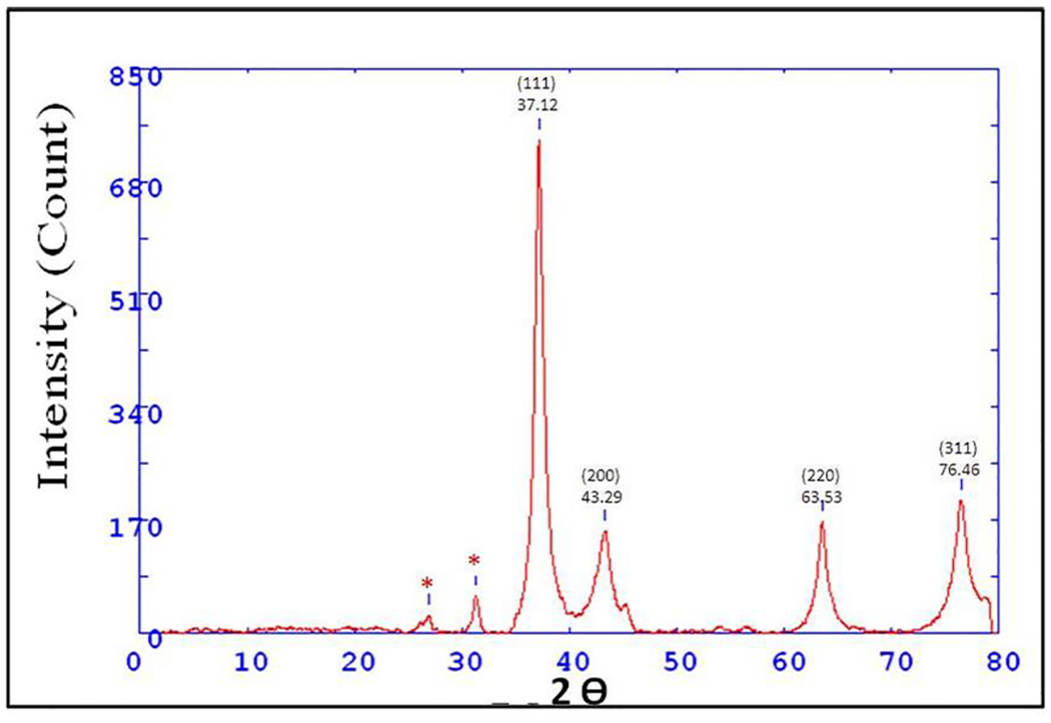
X-ray diffraction patterns of green AgNPs.

XRD confirms the crystalline nature of the nanoparticles. The XRD spectra demonstrated numbers of Bragg reflections that might be indexed on the basis of face-centered cubic structure of AgNPs. The comparison of synthesized AgNPs XRD spectrum with the standard showed crystalline nature of nanoparticles. As evidenced by the peaks at 2θ values of 37.12°, 43.29°, 63.53° and 76.46° corresponding to (111), (200), (220) and (311) Bragg reflections, respectively, which may be indexed based on the face-centered cubic structure of silver as shown in Fig 3.

#### Functional groups detection using FTIR spectra analysis

**Fig 4:**
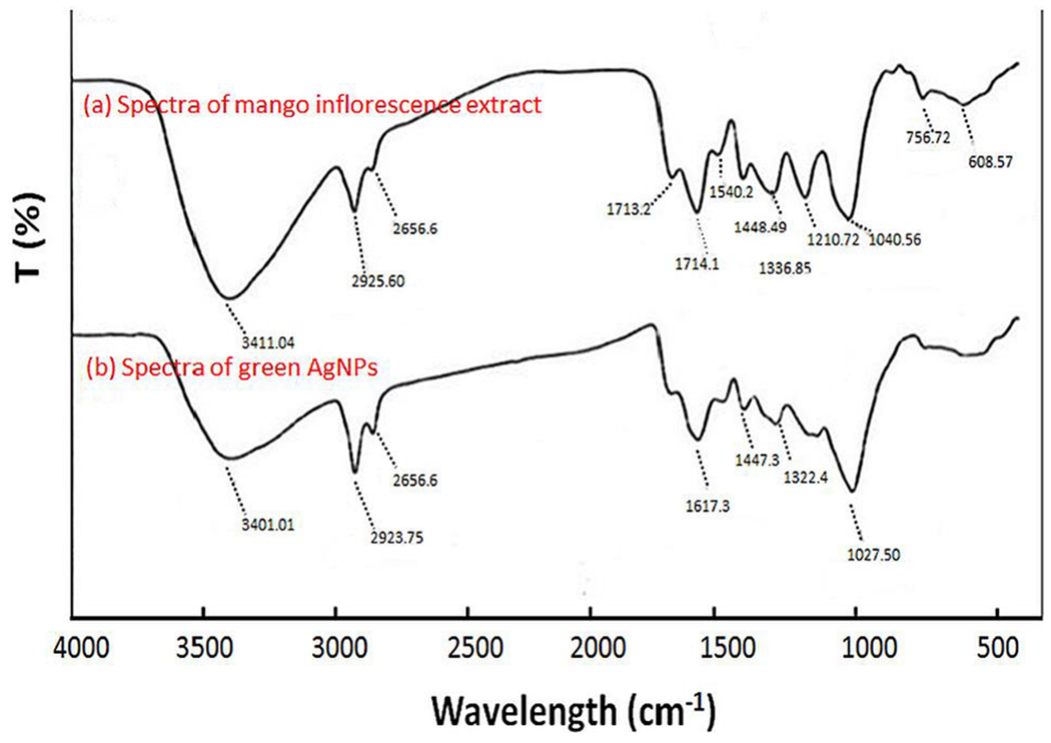
FTIR spectra (a) Aqueous plant extracts (b) AgNPs spectra.

Biological functional group involvement in nanoparticles formation was confirmed by FTIR technique. The FTIR data reveals that number of functional biological group responsible for stabilization of nanoparticles, which acts as capping or stabilizing agents. The FTIR spectra helps to predict the presence of possible biomolecules that are involved in reducing and stabilization of silver ions (Ag^+^) to silver nanoparticles (Ag°) present in aqueous extract (Fig 4). The band at 3405 cm^−1^ (shifted to 3401 cm^-1^ in AgNPs) was responsible for O-H stretching [21]. The band 2928 cm^−1^ was due to the presence of aldehyde C-H stretching and was shifted to lower frequency (2925 cm^−1^) in AgNPs, when compared with the extract. A peak was observed around 2856 cm^−1^ that could be assigned to the C–H stretching vibration and same peak was observed in AgNPs spectra. Peak at (C-O stretching) at 1716cm^−1^ present in extract was absent in AgNPs as it has been consumed in reduction process. The band 1041 cm^−1^ is responsible for C-O-C stretching, which could be attributed to the reduction of Ag^+^ because the band was shifted to 1027 cm^−1^ in AgNPs, it has been previously confirmed by FTIR spectra [22]. The band 1621 cm^−1^ in extraction was due to presence of amide I vibrations and this band was shifted to 1617 cm^−1^ in AgNPs because of the proteins that possibly has bound to AgNPs through the amine groups. The band at 1540 and 1454cm^−1^ assigned to the methylene scissoring vibrations from the proteins and 1210cm^-1^ corresponding to -C-N- bond stretching of amines were present in extract and absent in AgNPs.

### Assessment of antibacterial activity

MIC was recorded as the lowest concentration at which no visible growth was observable (Table 1). Green AgNPs and chemically synthesized AgNPs inhibited growth of Gram negative bacteria at 8 μg ml^-1^ and 16μg ml^-1^ respectively. Gram positive bacterial growth was inhibited at 32 μg ml^-1^ and 64 μg ml^-1^ by green AgNPs and chemically synthesized respectively. Green AgNPs inhibited bacterial at lower concentration as compared to chemically synthesized AgNPs.

**Table 1:** MICs and MBCs values of chemically synthesized and biogenic AgNPs.

Bacteria Name	MICs (μg ml^-1^) of green AgNPs	MICs (μg ml^-1^) of chemically synthesized AgNPs	MBCs (μg ml^-1^) of green AgNPs	MBCs (μg ml^-1^) of chemically synthesized AgNPs
Gram Negative				
*E*. *coli*	8	32	16	64
*P*. *aeruginosa*	8	32	16	64
*K*. *pneumoniae*	8	32	16	32
Gram Positive				
*S*. *mutans*	16	64	32	128
*S*. *aureus*	16	64	32	128

### Effect of AgNPs on growth of *E*. *coli* and *S*. *mutans*

**Fig 5:**
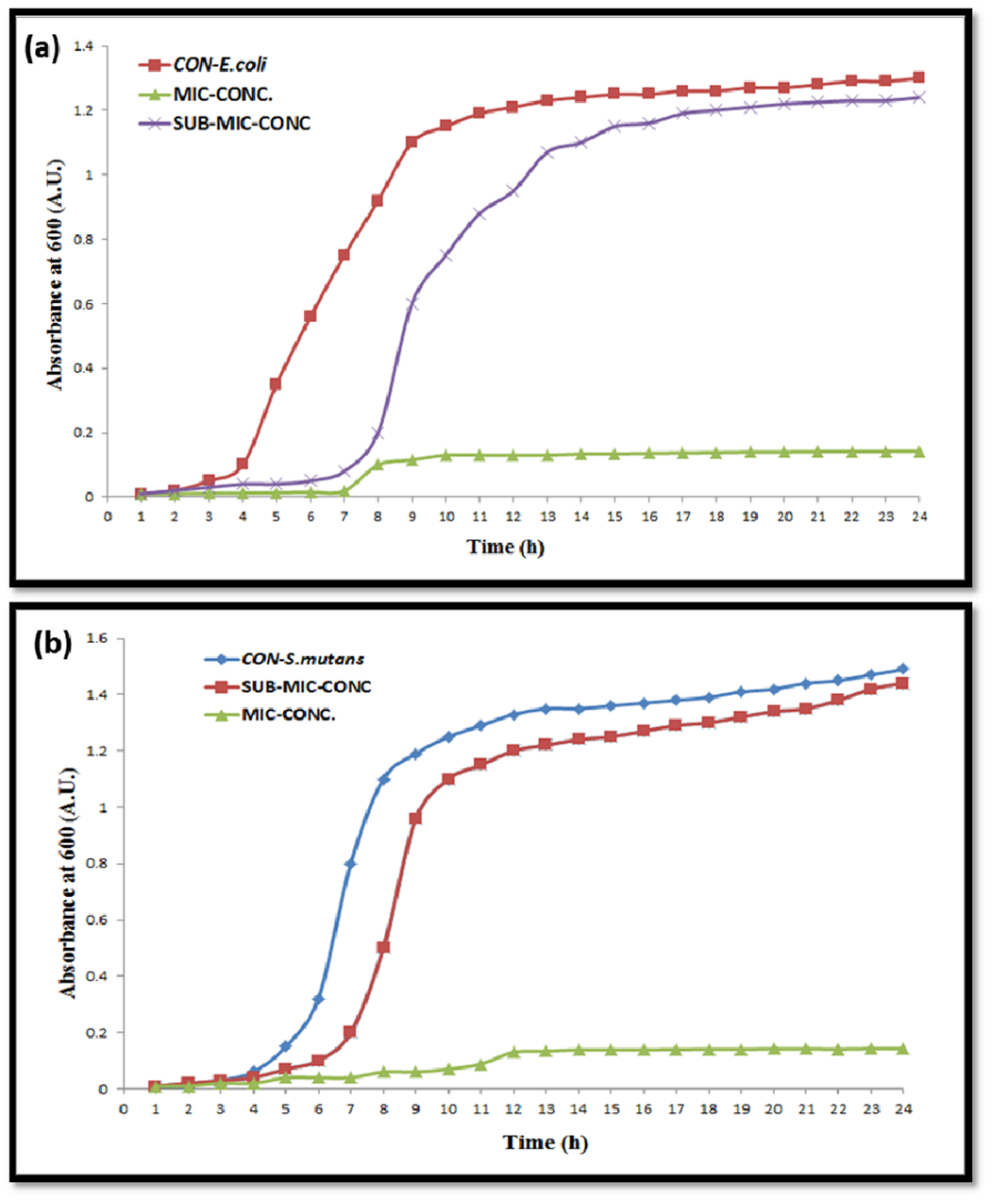
Growth curves of bacteria under the influence of AgNPs. (a) *E*. *coli* (b) *S*. *mutans*.

The effect of the AgNPs on the growth of *S*. *mutans* and *E*. *coli* cells is shown in Fig 5. No significant change was observed in the growth pattern of the control and treated bacteria at sub-MIC concentrations. The growth of *S*. *mutans* and *E*. *coli* was totally inhibited at their MIC concentrations. Hence, it was clear that AgNPs was inhibiting growth at MIC but not at sub-MIC concentration (Fig 5).

### Antibiofilm activity of green AgNPs confirmation using crystal violet assay, scanning electron microscopy and confocal microscopy

**Fig 6:**
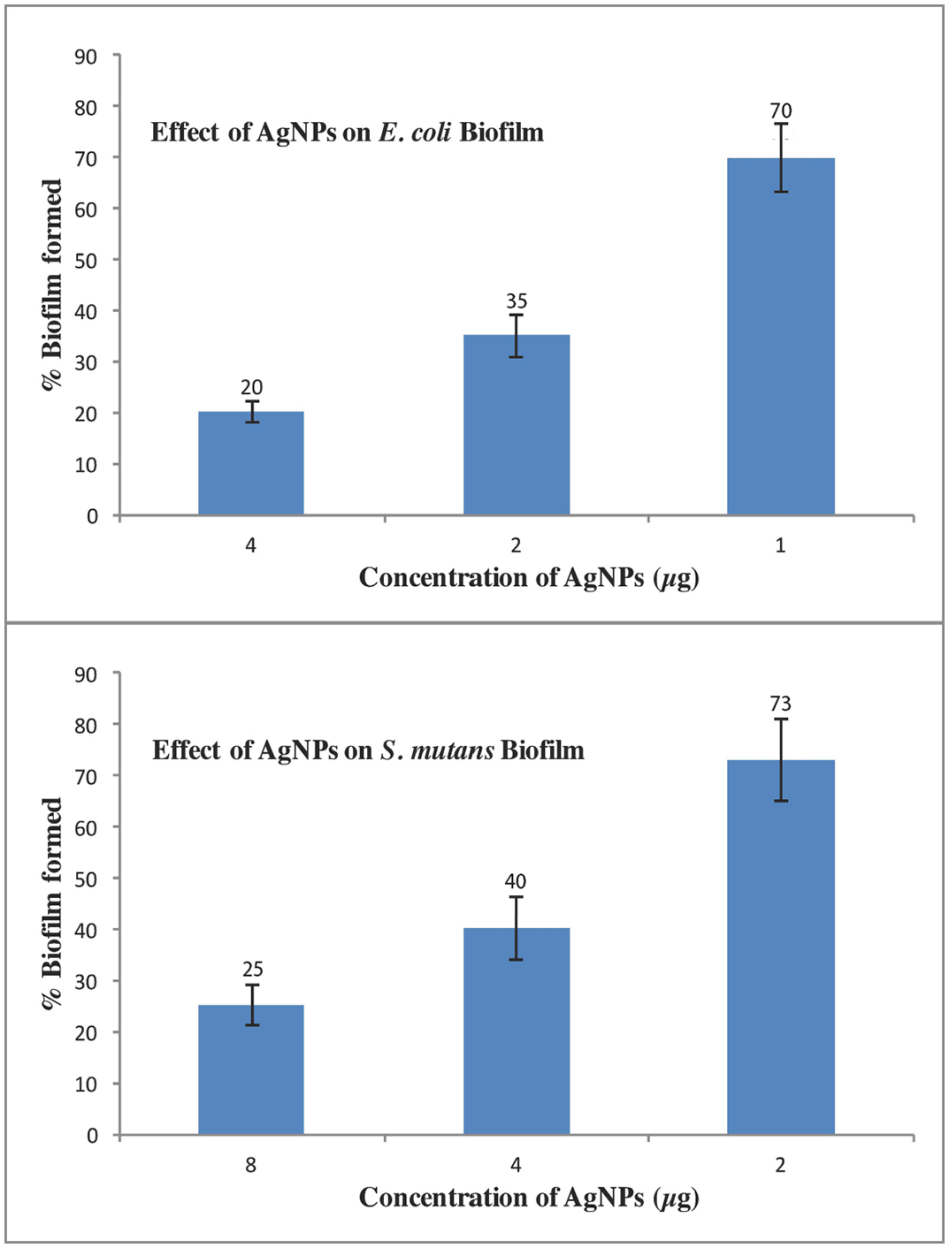
AgNPs antibiofilm activity against biofilms of *E*. *coli* and *S*. *mutans*. Error bars represent standard deviations of triplicate incubations.

**Fig 7:**
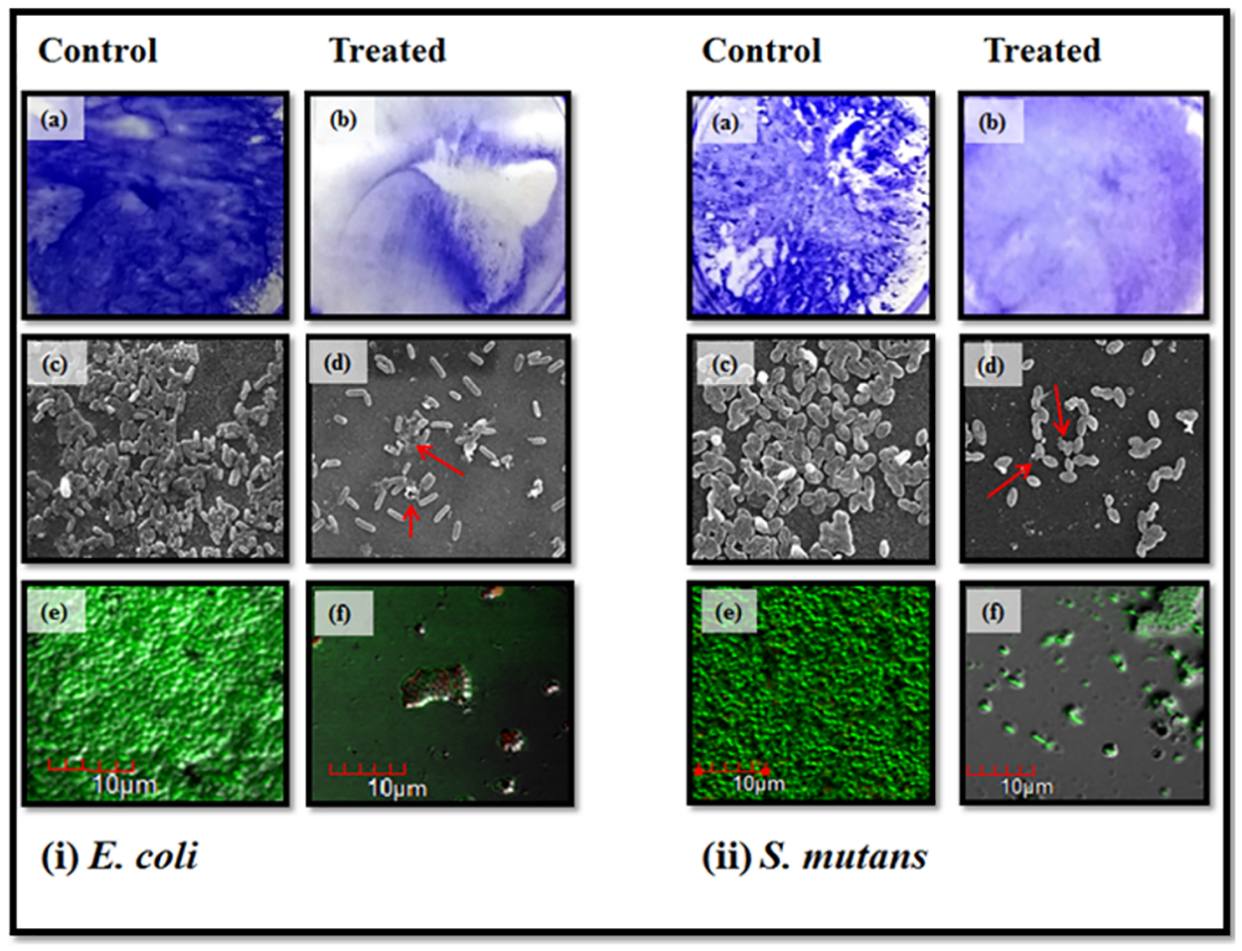
Visualization of the biofilm inhibition [i] Biofilms of *E*. *coli*. (a) & (b) images displays control and treated biofilm stained by crystal violet. (c) & (d) images shows the difference in biofilm after treatment as visualized by the SEM. Red arrows indicate the damage in the bacterial cells due to action of AgNPs. (e) & (f) are CLSM images of the control and treated biofilms. **[ii]** Biofilms of *S*. *mutans* (a)& (b) images displays control and treated biofilm stained by crystal violet. (c) & (d) images shows the difference in biofilm after treatment as visualized by the SEM. Red arrows indicate the damage in the bacterial cells due to action of AgNPs. (e) & (f) are CLSM images of the control and treated biofilms.

AgNPs inhibited biofilm formation in a dose-dependent manner. Crystal violet staining assay showed that biofilm of *E*. *coli* was suppressed by 80% when sub-MIC (4 μg ml^-1^) concentration of AgNPs was applied (Fig 6). In case of *S*. *mutans* these particles checked about 75% biofilm formation at sub-MICs concentration (μg ml^-1^). Further, visualization of control and treated biofilms using SEM and CLSM microscopy also confirmed a significant reduction in the biofilm of both *E*. *coli* and *S*. *mutans* on treatment with sub-MICs AgNPs (Fig 7).

### Detection of the protein and reducing sugar leakage from treated cells

**Fig 8:**
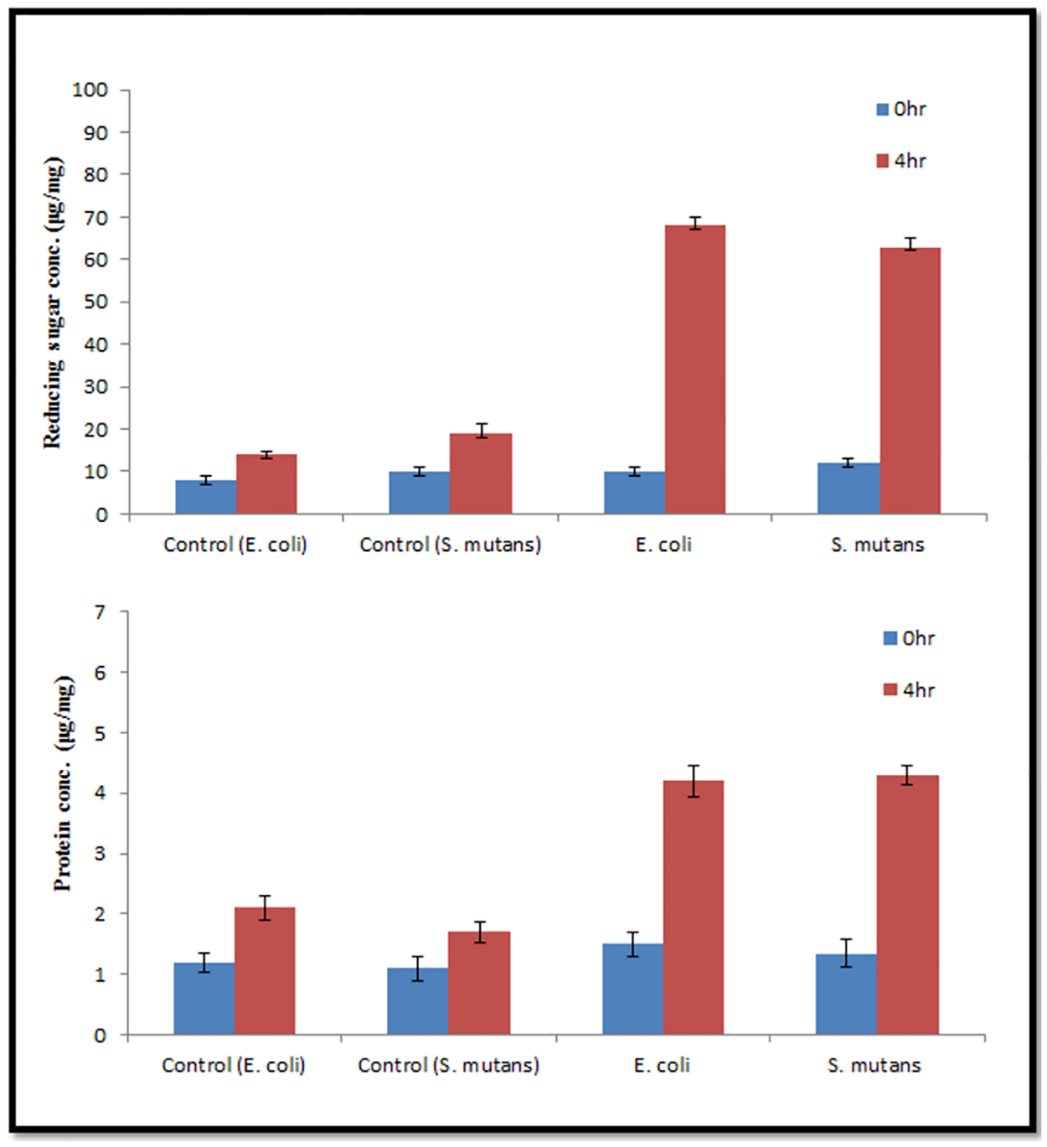
Leakage of reducing sugars (a) and proteins (b) from *E*. *coli* and *S*. *mutans* cells treated with AgNPs (Concentration of AgNPs is in μg ml^-1^). Error bars represent standard deviations of triplicate incubations.

Leakage assay reveled that there was three and two-fold increase in the amount of reducing sugars and the proteins after 4 h of incubation in treated samples compared to control. These results positively support the antibacterial tendency of these particles through lysing the bacterial cells. Untreated samples were taken as control and showed infinitesimal increase in the leaking products as shown in Fig 8.

### Interaction of AgNPs with bacterial cells

**Fig 9:**
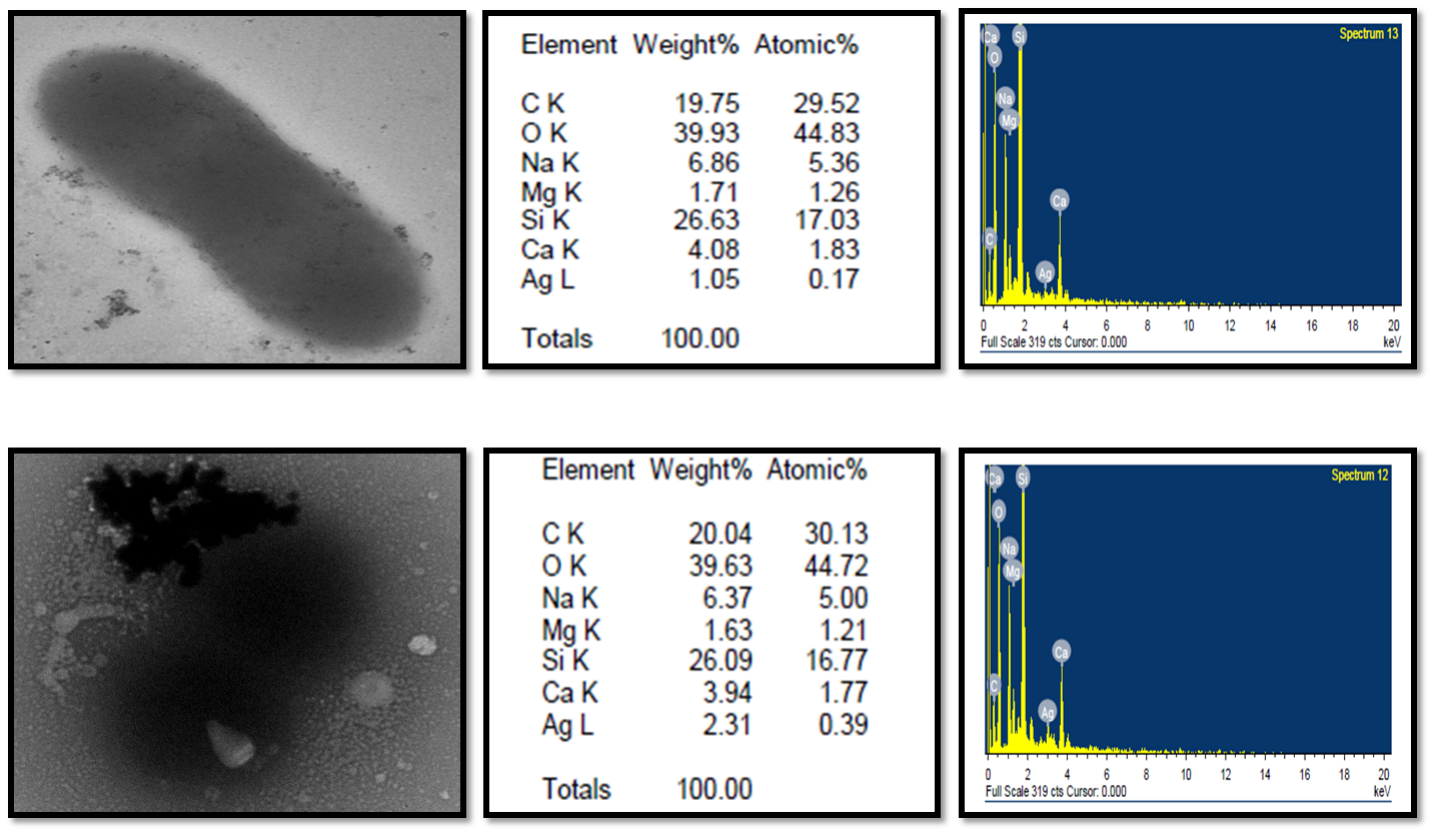
TEM images of bacterial cells treated with AgNPs and their EDX spectra.

TEM images confirmed that these AgNPs were found to be attached to the bacteria surface. The attached particle elemental identity was confirmed using EDAX, and it was clearly evident that these particles were made of silver. The AgNPs and bacterial attachment is shown in Fig 9. Most of the particles were seen attached to the surface.

### Detection of reactive oxygen species (ROS) and plasmid DNA cleavage activity

**Fig 10:**
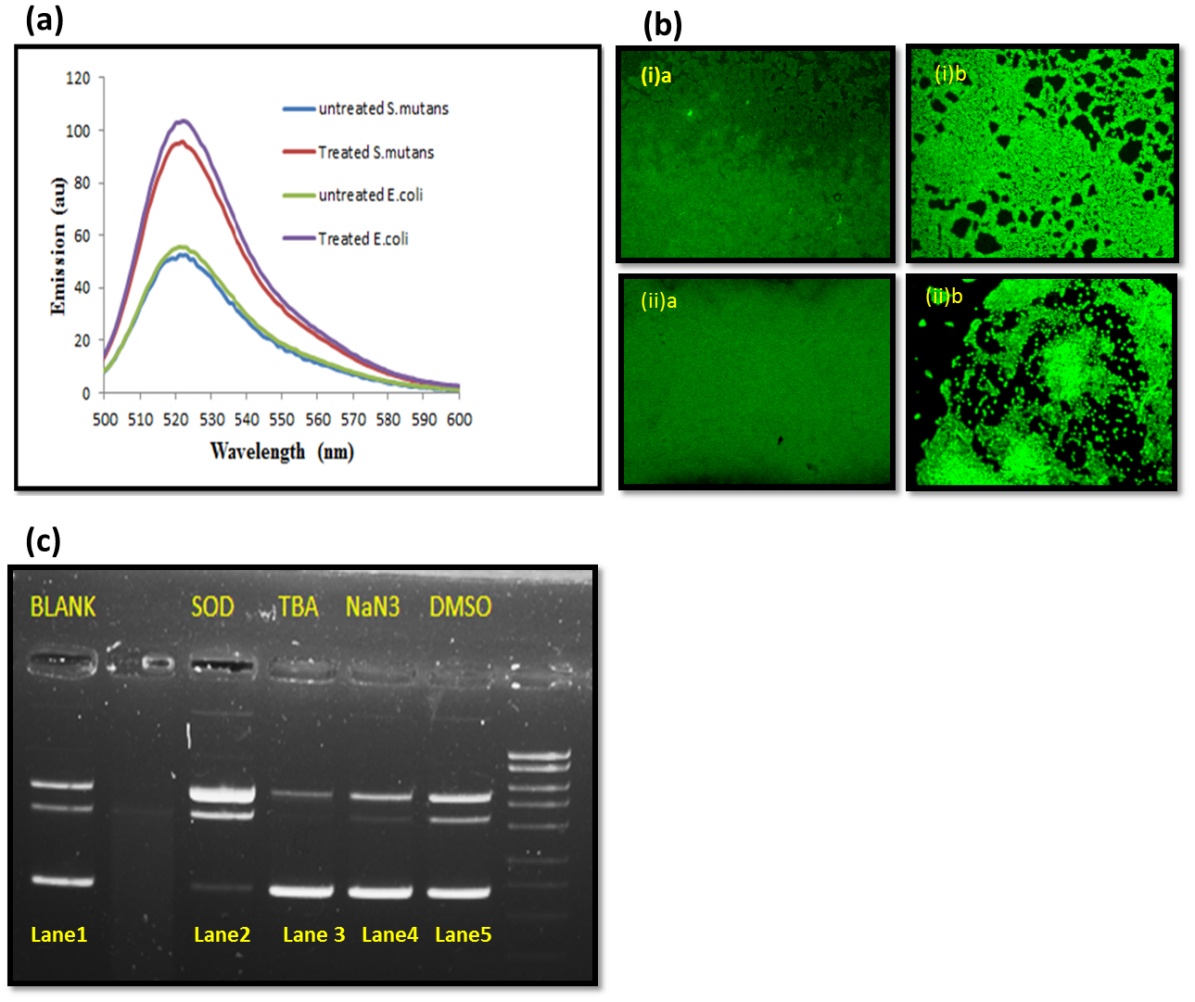
Reactive oxygen species experiments. (a) ROS production in presence of AgNPs in (a) *E*. *coli* (b) *S*. *mutans*. Error bars represent standard deviations of triplicate incubations. (b) Visualization of ROS generation by DCFH-DA green fluor probe in nanoparticle treated bacterial biofilms. AgNPs show the significant increment of green color fluorescence (ROS specific DCFH-DA probed) during 60 min incubation as compared to control bacterial biofilms. (i)a *S*. *mutans* control biofilm (i)b *S*. *mutans* treated biofilm. (ii)a *E*. *coli* control biofilm (ii)b *E*. *coli* treated biofilm. (c) Plasmid (DNA) cleavage assay in presence AgNPs (1.5 μg ml^-1^) and various free radical scavengers (DMSO, NaN3, TBA, and SOD). Lane 1, 3, 4 and 5 show the intact plasmid DNA; Lane 2 shows the cleaved plasmid DNA.

ROS production detection results showed green nanoparticles produced ROS when they were incubated with bacterial cells for 4h. It was clear that ROS was being produced when these green AgNPs were in contact with bacterial cells and increasing contact time increased in ROS concentration. Amount of ROS increased several times compared to the control for both Gram positive and Gram negative bacteria. Higher ROS was detected in Gram negative treated bacterial cells than treated Gram negative bacterial cells (Fig 10a). Treated bacterial biofilms confocal micrograph showed enhanced ROS generation (DCFH-DA) in comparison to normal ROS generation in control biofilms (Fig 10b). Plasmid nicking assay done in the presence of the various inhibitors showed that DMSO, TBA and NaN_3_ inhibited the cleavage action of AgNPs. SOD did not alter the cleavage action of AgNPs action. It clearly indicated that AgNPs cleavages plasmid mainly by OH° and ^1^O_2_ action with little involvement of superoxide ions (Fig 10c).

### Biofilm inhibition assay on catheter

**Fig 11:**
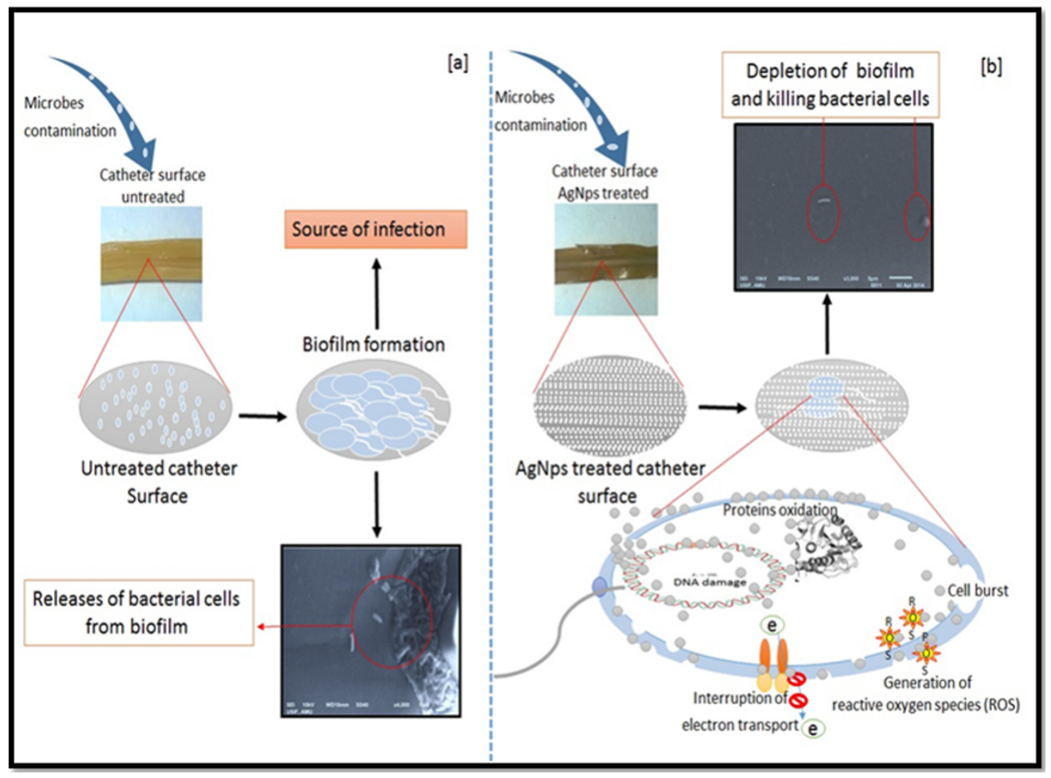
Graphical presentation of the anti-biofilm potency of the nanoparticles on catheter surface using scanning electron microscopy. (a) Untreated catheter incubated bacterial biofilm (b) nanoparticles treated catheter showing inhibition of biofilm on nano-modified surface.

Suppression of bacterial biofilm from the surface of AgNPs coated catheter pieces was observed by SEM. Hence, the release of adsorbed and absorbed nanoparticles from the catheter surface restricted the formation of bacterial biofilm. In this study, *E*. *coli* has been selected as model bacteria for biofilm inhibition on catheter tube (Fig 11).

### AgNPs cytotoxicity

**Fig 12:**
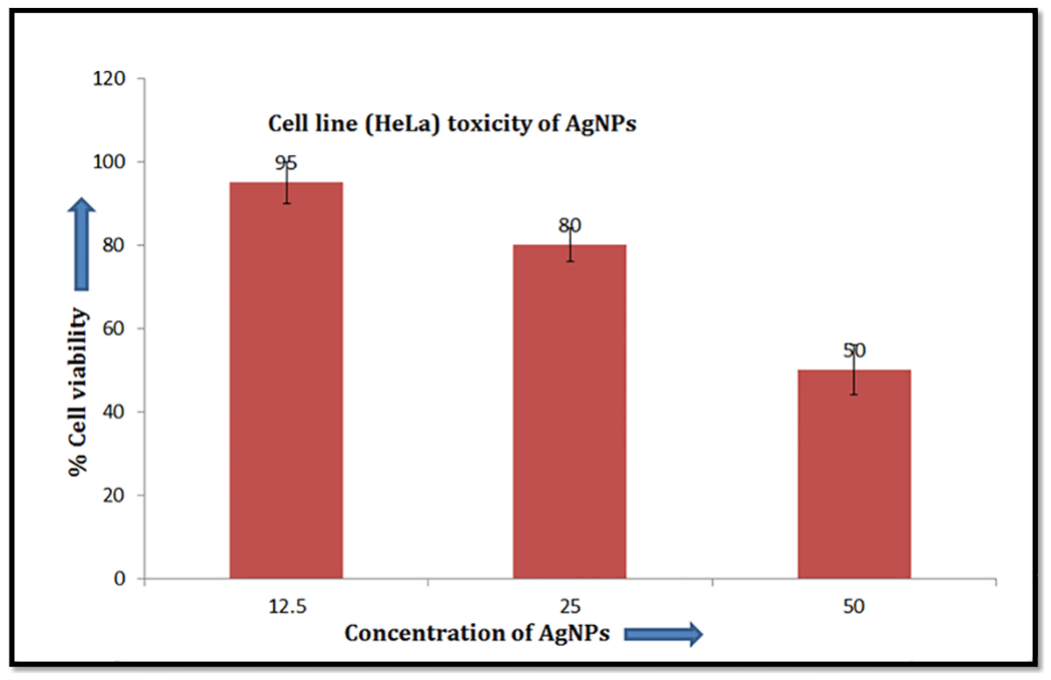
Cell line (HeLa) toxicity assessment of the AgNPs. Error bars represent standard deviations of triplicate incubations.

The toxicity of biogenic AgNPs from mango inflorescence was evaluated *in vitro* against HeLa cell line at 12.5 μg ml^-1^, 25 μg ml^-1^ and 50 μg ml^-1^ concentrations by MTT assays. The cells treated with 12.5 μg ml^-1^ were observed with negligible cellular toxicity. But higher AgNPs concentration such as 25 and 50 μg ml^-1^ caused 50 and 80% cell viability respectively (Fig 7). The IC_50_ of these nanoparticles was found to be 50 μg ml^-1^ for HeLa cell line. AgNPs showed inconsiderable cell toxicity of the at the MIC concentrations although its effect was dose dependent (Fig 12) and similar results were also reported by Arokiyaraj et al [23].

## Discussion

Singh *et al*. (2010) reported mango inflorescence as an effective antimicrobial agent [24]. In this study mango inflorescence an aqueous extract has been used as a reducing and stabilizing agent for the synthesis of stable AgNPs (Fig 1). The biosynthesized AgNPs were found to be stable for long period (>90 days) of time. The long-term stability of the AgNPs solution may be due to presence of small peptides and other proteins in the mango inflorescence extract working as capping agents for AgNPs. Further, we observed the function of time for the synthesis of AgNPs and the results advocate that the intensity of this peak increases with an increase in the reaction time. As, the intensity of the surface plasmon peak is directly proportional to the density of the NPs synthesis in solution [25]. The shape and structure of the nanoparticles has varying dimensions such as spherical, quasi-spherical, triangular and pentagonal as revealed by FESEM and TEM (Fig 2). The reasons for this heterogeneous particles formation phenomenon may be due to fast utilization of capping molecules and particles formed later were left with less capping molecules and hence facing a condition of thermodynamic instability. Particles formed later develops shapes like triangle or pentagon having smooth angle to minimize high surface energy [4, 26]. TEM images clearly indicated that these particles were mono-dispersed in nature and retained their structure even after a long period. Crystalline structure of AgNPs were analyzed using XRD analysis and by peak matching to related AgNPs. Biogenic AgNPs XRD spectra showed two extra peaks, which are denoted by stars and indicate the presence of biological moieties in the amorphous crystalline structure (Fig 3). Presence of such peaks in XRD of green nanoparticles was in total accordance with previous reports [4]. Biological functional groups involvement in nanoparticles formation was confirmed by FTIR (Fig 4) and these functional biological groups acts as capping or stabilizing agents [27].

Green AgNPs were found to be three times more efficient in antibacterial activity compared to chemically synthesized AgNPs. Gram negative bacteria were more effectively killed by green AgNPs in comparison to Gram positive bacteria (Table 1). The green synthesized AgNPs using peel of banana *Medicago sativa* and *Sesuvium portulacastrum* L. callus leaf displayed similar enhanced bactericidal activity results [28]. Multiple layer of peptidoglycan in Gram positive bacterial cell makes it a tough structure which restricts penetration of the AgNPs [29, 30]. But, Gram negative cell wall is composed of single or double layer peptidoglycan which makes it more prone to AgNPs attack [29, 30]. MBCs results confirmed the same trend as exhibited by MICs. The growth curve assay showed that these AgNPs were not effecting the bacterial growth pattern at sub-MICs (Fig 5) and AgNPs may be inhibiting virulence factors of the microbes without affecting bacterial viability at sub MIC concentrations.

Crystal violet assay results indicate that biofilms of both bacteria (*E*. *coli* and *S*. *mutans*) were inhibited with no major difference in the comparative antibiofilm activity (Fig 6). SEM and CLSM also confirmed that the biofilms of both the bacteria were inhibited in presence of green AgNPs at sub-MIC concentrations. It was clear from SEM and TEM microscopy images that biofilms are totally shattered and no bacterial cell clumping is observable (Fig 7). Further, the nanoparticle treated catheter resisted the formation of biofilm onto their surface (Fig 12). Hence, these green AgNPs can also be used to adhere the catheter surface and do not lose their activity even after immobilization to surfaces.

Scanning electron microscopy analysis of AgNPs treated bacterial cells revealed that these cells were having abnormal cell shape with pits and bulge present in the cell surface (Fig 7). This may have occurred due to AgNPs accumulation in bacterial membrane which leads to change in membrane potential. So, change in membrane potential promotes pit formation in membrane and further cell lysis leading to cell death [31].

Protein and reducing sugar leakage assay from the cells signifies that green AgNPs nanoparticles act on bacterial cells and lyse them or change their membrane permeability (Fig 8). Green nanoparticles have ability for faster and stronger attachment to the bacterial cell membrane in comparison to chemically synthesized nanoparticles which supports its better antibacterial action [22]. TEM was utilized to view the nature of interaction between AgNPs and bacterial cells. It was evident from images that AgNPs were mainly interacting with the bacterial cell surface (Fig 9). But, still one question was unanswered that how these AgNPs perform antibacterial and antibiofilm action? It was clearly visible from TEM and SEM images that there was little or no physical damage as most of the cells were found intact (Figs 7, 9 and 12).

So, we performed cellular ROS detection assays. The bacterial cells and AgNPs were incubated for 12 h and ROS generation was observed (Fig 10a). It was detected that interaction of AgNPs with bacterial cells resulted in ROS production. Gram negative bacteria produced more ROS than Gram positive bacteria in comparison to their controls (Fig 10a). ROS production results falls in accordance with the fact that thick Gram positive cell wall restricts AgNPs internalization in comparison to Gram positive bacteria and hence generating less ROS. The ROS production leads to bacterial membrane distortion or bacterial cell lysis which ultimately leads to cell death. ROS also causes denaturation of proteins and damage to other macromolecules which caused cell death and improper bacterial virulence factor expression including biofilm inhibition [27]. CLSM images revealed that AgNPs treated biofilms showed more than twice of ROS generation than untreated biofilms (Fig 10b). ROS generation damages the biofilm’s integrity and architecture by breaking the biofilm biomatrix and its constituent biomolecules. The biofilm matrix contains eDNA, protein and polysaccharides which are easily damaged by ROS action [32].

Plasmid DNA cleavage reactions were done in the presence of various free radical scavengers to know the types of free radical involved DNA cleavage or degradation. Hydroxyl free radical scavengers such as DMSO and TBA (tetra-butyl alcohol) and ^1^O_2_ scavenger, NaN_3_ reduced plasmid DNA cleavage activity which was indicative of the involvement of OH° and ^1^O_2_ radicals in the cleavage process. Moreover, superoxide anion free radical scavenger, such as SOD showed no inhibition of plasmid cleavage. So, it was clear that OH° and ^1^O_2_ radicals were mainly responsible for the plasmid nicking in the assay (Fig 10c). Predicted methods of bacteria inhibition by AgNPs includes binding on sulfur rich membrane proteins and DNA and ROS mediated inhibition of cell cycle. It is well known that extracellular DNA is very important in the early stages of the biofilm formation and DNA is damaged by nanoparticles as proved above and thus it disturbs the biofilm structure and integrity. It can be deduced from above discussion that these green AgNPs generate hydroxyl ions and singlet oxygen reactive species on interaction with bacterial surface, which further leads to bacterial cell wall and DNA breakdown.

## Conclusion

This is an effective and eco-friendly approach to develop nanoparticles with multiple applications from *Mangifera indica* inflorescence extract. The characterized particles inhibited bacterial growth and biofilm at sub-MIC concentrations. SEM, TEM and EDAX showed that AgNPs were acting on the bacterial surface and inhibiting its biofilm. This interaction produces certain specific ROS species which leads to bacterial cell death and obliteration in biofilms. AgNPs coated catheter tubes also resisted the bacterial growth on its surface. Green AgNPs made from *Mangifera indica* apart from being strong antimicrobials also showed quality for a future antibiofilm agent to eradicate biofilms formed by pathogenic microbes by the involvement of ROS.
